# The early growth response protein 1-miR-30a-5p-neurogenic differentiation factor 1 axis as a novel biomarker for schizophrenia diagnosis and treatment monitoring

**DOI:** 10.1038/tp.2016.268

**Published:** 2017-01-10

**Authors:** S Liu, F Zhang, Y Y Shugart, L Yang, X Li, Z Liu, N Sun, C Yang, X Guo, J Shi, L Wang, L Cheng, K Zhang, T Yang, Y Xu

**Affiliations:** 1 Department of Psychiatry, First Hospital of Shanxi Medical University, Taiyuan, China; 2 Wuxi Mental Health Center, Wuxi, China; 3 Unit on Statistical Genomics, Intramural Research Program, National Institute of Mental Health, National Institutes of Health, Bethesda, MD, USA; 4 Department of Biochemistry and Molecular Biology, Shanxi Medical University, Taiyuan, China; 5 First Clinical Medical College of Shanxi Medical University, Taiyuan, China

## Abstract

To date, diagnosis of schizophrenia is still based on clinical interviews and careful observations, which is subjective and variable, and can lead to misdiagnosis and/or delay in diagnosis. As early intervention in schizophrenia is important in improving outcomes, objective tests that can be used for schizophrenia diagnosis or treatment monitoring are thus in great need. MicroRNAs (miRNAs) negatively regulate target gene expression and their biogenesis is tightly controlled by various factors including transcription factors (TFs). Dysregulation of miRNAs in brain tissue and peripheral blood mononuclear cells (PBMNCs) from patients with schizophrenia has been well documented, but analysis of the sensitivity and specificity for potential diagnostic utility of these alternations is limited. In this study, we explored the TF-miRNA-30-target gene axis as a novel biomarker for schizophrenia diagnosis and treatment monitoring. Using bioinformatics analysis, we retrieved all TFs that control the biogenesis of miRNA 30 members as well as all target genes that are regulated by miRNA-30 members. Further, reverse transcription-quantitative PCR analysis revealed that the early growth response protein 1 (EGR1) and miR-30a-5p were remarkably downregulated, whereas neurogenic differentiation factor 1 (NEUROD1) was significantly upregulated in PBMNCs from patients in acute psychotic state. Antipsychotics treatment resulted in the elevation of EGR1 and miR-30a-5p but the reduction of NEUROD1. Receiver operating characteristic analysis showed that the EGR1-miR-30a-5p-NEUROD1 axis possessed significantly greater diagnostic value than miR-30a-5p alone. Our data suggest the EGR1-miR-30a-5p-NEUROD1 axis might serve as a promising biomarker for diagnosis and treatment monitoring for those patients in acute psychotic state.

## Introduction

Schizophrenia is a debilitating mental disorder with an overall prevalence estimate of ~4.0 per 1000 people.^1, 2^ This major psychotic disorder is characterized by early adulthood onset and complex clinical symptoms related to neurocognitive and neurophysiological dysfunction. Although genetic involvement in the pathogenesis is implicated,^3, 4, 5, 6, 7, 8, 9, 10, 11^ the etiology of schizophrenia remains to be elucidated.

Currently, diagnosis of schizophrenia is based on clinical interviews and careful observations. These descriptive methods are subjective and variable, which can lead to delay in diagnosis and/or misdiagnosis.^12^ As early intervention in schizophrenia is important in improving outcome,^13, 14^ objective tests that can be used for schizophrenia diagnosis or medication response monitoring are thus in great need to enable effective treatment.^12, 15, 16^ To date, studies have particularly explored peripheral blood, due to its accessibility and ease in procurement, for the identification of biomarkers for schizophrenia.^16, 17, 18, 19, 20, 21, 22^

MicroRNAs (miRNAs) are a class of small non-coding RNA molecules (~19–22 nt in length) that negatively regulate gene expression at the post-transcriptional level.^23, 24^ They bind to the 3′-untranslated region of target messenger RNAs (mRNAs) through complementarity, resulting in mRNA degradation and/or translational inhibition. It is known that miRNAs control a wide range of biological functions and processes.^25, 26^ Alternation of miRNA expression has been implicated in various diseases including schizophrenia.^27, 28, 29, 30, 31^ Perkins *et al.*^29^ have shown that miR-30 members, that is, miR-30a-5p, miR-30b, miR-30d and miR-30e are downregulated in the prefrontal cortex of subjects with schizophrenia compared with healthy subjects. MiR-30b expression is significantly reduced in the cerebral cortex of female subjects with schizophrenia.^31^ Microarray analysis has shown that 33 miRNAs including miR-30d and miR-30e are reduced in peripheral blood mononuclear cells (PBMNCs) isolated from patients with schizophrenia.^17^ It is known that miRNA biogenesis is under tight control by a variety of regulators including transcription factors (TFs) that act at the transcription level.^32, 33^ There is evidence indicating that altered TF can result in changes in miRNA expression, which is responsible for disease phenotypic variations.^34^ In view of several reports showing miR-30 dysregulation in schizophrenia,^17, 29, 31^ we hypothesized that the TF-miR-30-target gene axis might alter in schizophrenia, and that the changed axis might serve better as a biomarker than a single miRNA. This study was therefore conducted to investigate the potential of the TF-miRNA-30-traget gene axis in PBMNCs as a novel biomarker for schizophrenia diagnosis and treatment monitoring.

## Materials and methods

### Subjects

This study was approved by the Research Ethics Committee of the First Hospital of Shanxi Medical University. Written informed consent was obtained from healthy participants and a first-degree relative of patients. All participants were of unrelated Chinese Han nationality. To ensure adequate power to detect a pre-specified effect, the sample size was chosen using the Power and Sample Size Program (http://biostat.mc.vanderbilt.edu/PowerSampleSize). A total of 38 patients with schizophrenia in acute stage who had been drug-free for at least 3 months were recruited between March 2012 and February 2013. Clinical diagnosis was performed by at least two consultant psychiatrists independently according to the Diagnostic and Statistical Manual of Mental Disorders Fourth Edition (DSM-IV) criteria for SZ (https://justines2010blog.files.wordpress.com/2011/03/dsm-iv.pdf), relying on the Chinese Version of the Modified Structured Clinical Interview for DSM-IV TR Axis I Disorders Patient Edition (SCID-I/P,11/2002 revision).^35^ Those who were pregnant or had significant medical conditions, unstable psychiatric features (for example, suicidal feelings) or had a history of substance abuse or drug addiction within the previous 6 months were excluded.

The control group consisted of 50 healthy volunteers who were recruited from local communities or during routine health check-ups. All control subjects were assessed using the SCID-I/P, 11/2002 revision. Subjects with diagnosed diseases or a history of major psychiatric disorders or suicidal behavior were excluded, and those who had a first-degree relative with a history of severe mental disorder or suicidal behavior were also excluded.

### Medication and clinical assessment

All patients were treated with oral administration of one of the second-generation antipsychotics or atypical antipsychotics for 12 weeks. Eight patients were withdrawn from the study due to poor compliance. Therefore, only 30 patients completed the 12-week treatment as follows: 16 took risperidone (initial dose 2 mg, average dose 4.5 mg and dose range 2–6 mg); 7 took olanzapine (initial dose 5 mg, average dose 13 mg and dose range 5–20 mg); 3 took quetiapine (initial dose 100 mg, average dose 550 mg and dose range 100–800 mg); 2 took aripiprazole (initial dose 5 mg, average dose 12.5 mg and dose range 15–30 mg); and 2 took ziprasidone (initial dose 40 mg, average dose 135 mg and dose range 40–140 mg). The medication was taken once daily. For the first 1–4 weeks, the initial dose was increased to the curative dose. For the rest weeks, the maintenance dose was adjusted according to side effects and clinical assessment. If necessary, small doses of benzodiazepines were prescribed for agitation.

The clinical effects were assessed by trained and experienced psychiatrists with the Positive and Negative Syndrome Scale (PANSS) 25 before and after 12-week treatment. Altogether, the assessments contained 33 items, including 7 items for positive symptoms, 7 items for negative symptoms, 16 items for general psychopathology symptoms and 3 items for aggressiveness. Clinical improvement was defined as a PANSS reductive ratio >25%.

### Bioinformatic analysis

Bioinformatic analysis was performed to discover the potential TF-miRNA-30-traget gene axis. First, the TF-miRNA regulation database TransmiR (http://cmbi.bjmu.edu.cn/transmir) created by Wang *et al.*^36^ was screened. Subsequently, miRNA 30 targets were retrieved using the miRNA targets database R package multiMiR (http://multimir.ucdenver.edu), a comprehensive collection of predicted and validated miRNA target interactions and their associations with diseases and drugs.^37^

### Analysis of miRNA expression by reverse transcription-quantitative PCR

All blood samples were taken in the early morning between 0800 and 0900 hours. A total of 30 ml of peripheral blood was collected into K2-EDTA tubes (Beckton Dickinson, Franklin Lakes, NJ, USA), and PBMNCs were isolated using the Ficoll gradient protocol as described elsewhere.^38^ Total RNA from PBMNCs was extracted using the TRIzol reagent (Invitrogen Biotechnology, Shanghai, China) with on-column DNase I treatment as per the manufacturer’s instructions. After quantified with spectrophotometry, 1 μg of RNA was reverse-transcribed using the miScript Reverse Transcription kit (Qiagen China, Shanghai, China) according to the manufacturer’s instructions. Afterwards, 2 μl of reverse transcription product was used for measurement of miR-30 s with quantitative PCR using the miScript SYBR Green PCR kit (Qiagen China). PCR was performed on the 7900HT Sequence Detection System (Applied Biosystems, Waltham, MA, USA) as follows: step 1, 95 °C for 15 min; and step 2, 94 °C for 15 s, 55 °C for 30 s and 70 °C for 34 s. Step 2 was repeated for 40 cycles. The relative expression level of each individual miRNA after normalization to the small nuclear RNA U6 was calculated using the 2^−^^ΔΔCt^ method.

### Analysis of gene expression by reverse transcription-quantitative PCR

Reverse transcription with 2 μg of total RNA was done using a High Capacity RNA-to-cDNA Kit (Invitrogen Biotechnology) as per the manufacturer’s instructions. Quantitative PCR was performed using the primers listed in Table 1 and a SYBR®Select Master Mix (Invitrogen Biotechnology). PCR was run on the 7900HT Sequence Detection System as follows: step 1, 50 °C for 2 min; step 2, 95 °C for 2 min; and step 3, 95 °C for 15 s and 60 °C for 60 s. Step 3 was repeated for 40 cycles. Dissociation curves were set up for each reaction to examine the specificity of the amplification. The relative expression level of each individual gene after normalization to the glyceraldehyde 3-phosphate dehydrogenase gene was calculated using the 2^−^^ΔΔCt^ method.

### Receiver operating characteristic curve analysis

Receiver operating characteristic curves were drawn and the area under the curve (AUC) was measured to determine and compare the diagnostic values of single miRNA and the TF-miRNA target gene axis using the Statistical Package for Social Sciences version 17.0 (SPSS17.0; SPSS, Chicago, IL, USA).

### Statistical analysis

MiRNA and gene expression data were expressed as mean±s.e.m. Statistical analysis of the differences in miRNA and gene expression was performed by one-way analysis of variance. Further, two groups’ comparison was performed using the least-significant difference test. PANSS scores before and after treatment were tested by the paired *t*-test. The correlation of miRNA alternation with PANSS scores was tested by Pearson’s correlation test. The SPSS17.0 software was used for statistical analysis. *P*<0.05 was considered statistically significant.

## Results

### Study subjects

Preliminary data from 10 patients and 10 controls showed that the levels of *EGR1* and miR-30a-5p within each group were distributed with a respective s.d. of ~2.0 and if the true difference in the patient and control means is ~1.5 as observed in the preliminary study, according to the analysis with the Power and Sample Size Program, we would need to study at least 29 cases and 29 controls to be able to reject the null hypothesis that the means of the patient and control groups are equal with a probability (power) of 0.8. The type I error probability associated with this test of this null hypothesis is 0.05. The preliminary data of *NEUROD1* within each group were distributed with a respective s.d. of ~3.0 and if the true difference in the patient and control means is ~2.0 as observed in the preliminary study, we would need to study at least 36 cases and 36 controls to be able to reject the null hypothesis that the means of the patient and control groups are equal with a probability (power) of 0.8. The Type I error probability associated with this test of this null hypothesis is 0.05. Subsequently, 38 patients and 50 healthy individuals were recruited in this study. Over half of patients (20/38) had first-episode schizophrenia. Both groups had an age range of 19–60 years. The summarized demographics of all participants were listed in Table 2.

### Bioinformatics analysis results

**Figure 1:**
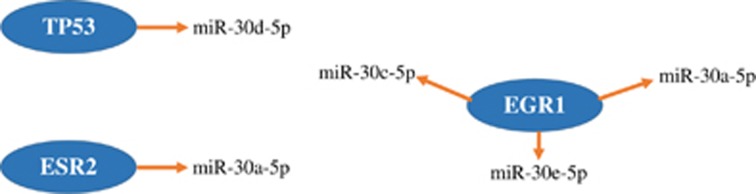
All transcription factors regulating miR-30 expression. Shown in this figure are those transcription factors identified from the database TransmiR that regulate the expression of miR-30 family members. EGR1, early growth response protein 1; ESR2, estrogen receptor 2; TP53, tumor protein 53.

Using the database TransmiR, we identified three TFs, that is, tumor protein 53 (*TP53*), estrogen receptor 2 (*ESR2*) and early growth response protein 1 (*EGR1*), that regulate the expression of miR-30 family members (Figure 1). Target genes of those miRNAs were further retrieved using the miRNA targets database R package multiMiR (Table 3).

### TF mRNA levels determined by reverse transcription-quantitative PCR

**Figure 2:**
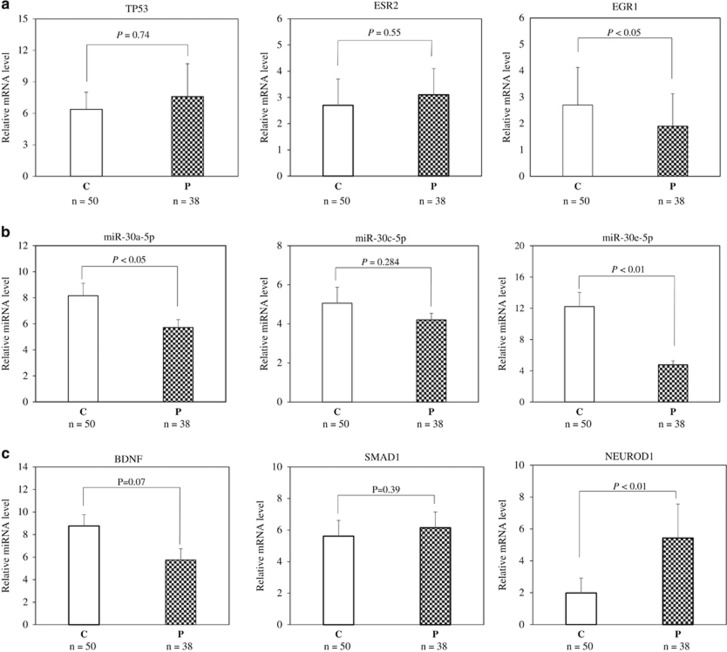
Reverse transcription-quantitative PCR analysis of the TF-miR30-target gene axis. The early growth response protein 1 *(EGR1)* messenger RNA (mRNA) level was found significantly lower in peripheral blood mononuclear cells (PBMNCs) from patients than in cells from healthy controls, whereas mRNA levels for tumor protein 53 *(TP53)* and estrogen receptor 2 *(ESR2)* in patients and healthy subjects were not significantly different (**a**). Levels of miR-30a-5p and miR-30e-5p in PBMNCs were found significantly lower in patients, whereas there was no significant difference in miR-30c-5p levels between the two groups (**b**). Neurogenic differentiation factor 1 *(NEUROD1)* expression in PBMNCs from patients was significantly increased compared with healthy controls, whereas the expression levels of brain-derived neurotrophic factor *(BDNF)* and mothers against decapentaplegic homolog 1 *(SMAD1)* were not significantly different between the patients and healthy subjects (**c**). C, healthy controls; P, patients.

mRNA levels of *TP53*, *ESR2* and *EGR1* in PBMNCs from patients and controls were determined by reverse transcription-quantitative PCR. The results showed that mRNA levels for *TP53* and *ESR2* in patients and healthy subjects were not significantly different (Figure 2a). In contrast, the *EGR1* mRNA level in PBMNCs from patients was 1.91±1.23, which was significantly lower than that in healthy individuals (2.70±1.43, *P*<0.05; Figure 2a).

### MiRNA and target gene mRNA levels determined by reverse transcription-quantitative PCR

Based on above findings, EGR1-regulated miRNAs, that is, miR-30a-5p, miR-30c-5p and miR-30e-5p, were further comparatively studied in PBMNCs from patients and healthy controls. As shown in Figure 2b, levels of miR-30a-5p and miR-30e-5p were found significantly lower in patients, whereas there was no significant difference in miR-30c-5p levels between the two groups. As shown in Table 3, only one gene, namely, the ubiquitin-conjugating enzyme E2I (*UBE2I*), was sorted out as the target of miR-30e-5p from the database R package multiMiR. A paucity of information on *UBE2I* role in schizophrenia is found in literature. Therefore, *UBE2I* was not further studied. Among the targets of miR-30a-5p, three genes, that is, brain-derived neurotrophic factor (*BDNF*), mothers against decapentaplegic homolog 1 (*SMAD1*) and neurogenic differentiation factor 1 (*NEUROD1*), all known to be associated with schizophrenia and mental disorders, were chosen for further analysis by reverse transcription-quantitative PCR. As shown in Figure 2c, *NEUROD1* expression in PBMNCs from patients was significantly higher than that in healthy controls (5.43±2.13 vs 1.98±0.94; patients vs controls, *P*<0.01), whereas there was no significant difference in *BDNF* and *SMAD1* mRNA levels between the two groups.

### Response of the EGR1-miR-30a-5p-NEUROD1 axis to pharmacotherapy

**Figure 3:**
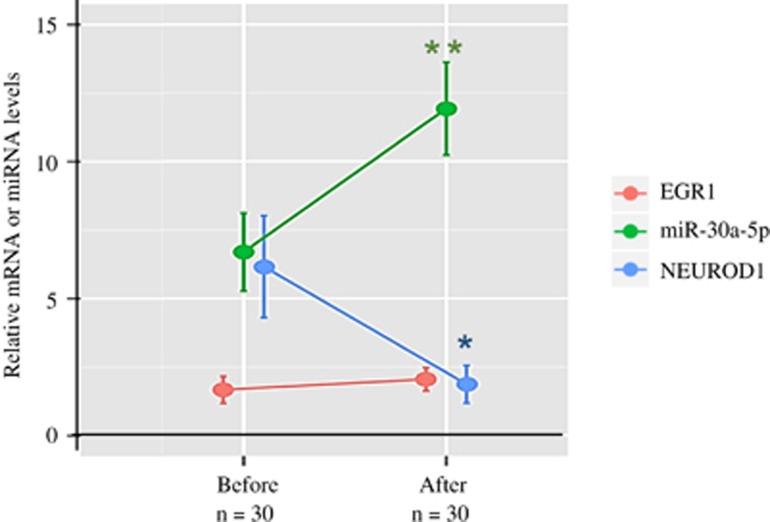
Response of the EGR1-miR-30a-5p-NEUROD1 axis to antipsychotics treatment. The early growth response protein 1 *(EGR1)* messenger RNA (mRNA) level was slightly increased after the treatment, which was not statistically significant. However, the treatment resulted in a significant elevation of miR-30a-5p levels and a significant reduction of neurogenic differentiation factor 1 *(NEUROD1)* mRNA levels, respectively. *P<0.05; **P<0.01.

Reverse transcription-PCR data demonstrated the alternation of the EGR1-miR-30a-5p-NEUROD1 axis in PBMNCs from patients, we then further examined the response of this axis to antipsychotics treatment. As shown in Figure 3, *EGR1* mRNA level was slightly increased after the treatment, which was not statistically significant (*P=*0.19). However, both miR-30a-5p and *NEUROD1* mRNA levels altered significantly after pharmacotherapy. The treatment resulted in a significant elevation of miR-30a-5p levels (*P*<0.01), and a significant reduction of *NEUROD1* mRNA levels (*P*<0.05), respectively.

### Correlation between miR-30a-5p elevation and symptom improvement

Symptom improvement was measured on the basis of the reduction of the PANSS score before and after treatment. After the 12-week medication, the PANSS total score and the cluster scores for positive symptoms, negative symptoms and general psychopathology symptoms were all significantly reduced (*P*<0.001; Table 4). The correlation between the reduction in the PANSS score and miR-30a-5p elevation was evaluated. A negative correlation was observed between the increase of miR-30a-5p and the reduction of negative symptoms (*r*=−0.363, *P*<0.05).

### Receiver operating characteristic analysis results

**Figure 4:**
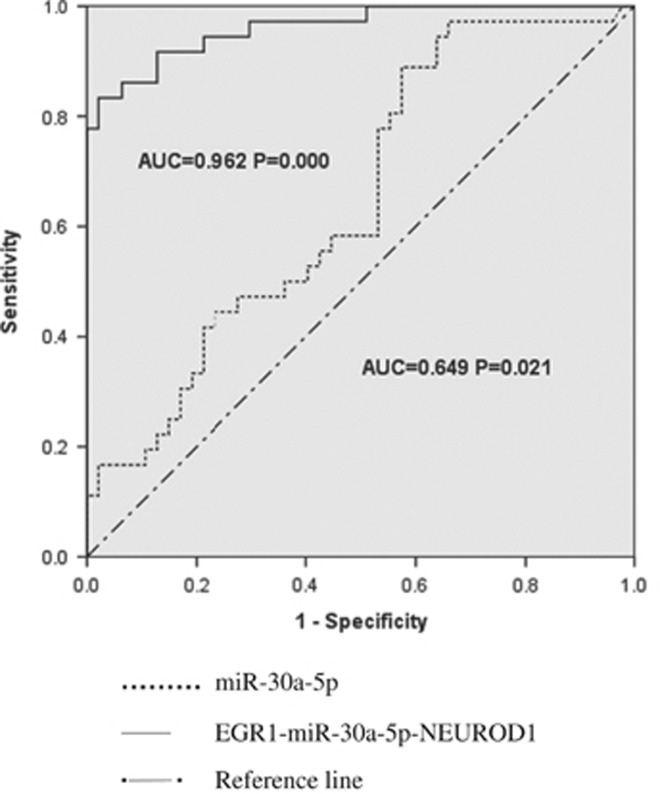
Receiver operating characteristic (ROC) curve analysis. ROC analysis showed an area under the curve (AUC) of 0.649 for miR-30a-5p alone and an AUC of 0.962 for the EGR1-miR-30a-5p-NEUROD1 axis, suggesting the EGR1-miR-30a-5p-NEUROD1 axis has a greater diagnostic value than miR-30a-5p alone.

Receiver operating characteristic analysis showed an AUC of 0.649 for miR-30a-5p (*P=*0.021, 95% confidence interval: 0.531–0.767) and an AUC of 0.962 for the EGR1-miR-30a-5p-NEUROD1 axis (*P*<0.021, 95% confidence interval: 0.924–0.999; Figure 4). Youden index (sensitivity+specificity−1) was further calculated to determine the optimal cutoff value of the EGR1-miR-30a-5p-NEUROD1 axis. The result demonstrated that the maximum Youden index was 0.812 when the cutoff achieved with sensitivity of 83.3% and specificity of 97.9%.

## Discussion

The importance of biomarkers in the context of schizophrenia diagnosis and treatment has been increasingly appreciated.^12, 15, 16^ Although the search for such biomarkers is challenging as schizophrenia is a complex and heterogeneous disorder, significant progress has been made using various biological approaches such as transcriptomics, proteomics, metabolomics, epigenetics and so on.^15^ Specifically, abnormalities at the molecular levels, for example, differentially expressed genes/miRNAs and aberrant signaling pathways, are discovered in the post-mortem brain tissue from patients with schizophrenia.^3, 4, 5, 6, 7, 8, 9, 10, 11, 29, 30, 31^ So far, the TF-miRNA-target gene axis has hardly been studied either as a factor in the pathogenesis of the disease or as a potential biomarker for schizophrenia. In view of the relevance of miR-30 members in schizophrenia, in this study, the TF-miR-30-target gene axis in PBMNCs was explored as a biomarker for schizophrenia diagnosis and treatment monitoring. Using bioinformatics tools combined with molecular biological approaches and statistical methods, we made the following findings: (1) the EGR1-miR-30a-5p-NEUROD1 axis altered in patients with schizophrenia; (2) the EGR1-miR-30a-5p-NEUROD1 axis responded highly to antipsychotics treatment; and (3) the EGR1-miR-30a-5p-NEUROD1 axis possessed significantly greater diagnostic value than miR-30a-5p alone.

Central nervous system can influence gene expression, metabolism and other activities in the peripheral blood cells via cytokines, neurotransmitters or hormones.^39, 40^ In addition, prenatal maternal infection is recognized as an important risk factor for schizophrenia, which may also leave immunologically relevant signatures in blood.^41, 42, 43, 44^ Therefore, blood-based schizophrenia markers have drawn substantial attention, as it is difficult to obtain the brain tissue from living people. Previous work has shown moderate decrease of plasma BDNF in patients. However, a meta-analysis reveals that plasma BDNF levels do not correlate with either the positive or negative symptom in schizophrenia patients, suggesting that plasma BDNF is unlikely a valid biomarker for the disorder.^18^ Increased serum inflammatory cytokines, for example, tumor necrosis factor, interleukin-1β, interleukin-6 and so on, are found in patients with schizophrenia, which however are also observed in those with bipolar disorder.^16^ Altered expression of mitochondrial complex I genes in whole-blood cells and platelets has been suggested as a potential peripheral marker for schizophrenia, although these genes are not responsive to neuroleptic treatment.^21, 22, 45^ Interestingly, the mRNA level of *NDUFV2*, one of the mitochondrial complex I genes, is positively correlated with positive symptoms in the first-episode schizophrenia patients,^45^ and complex I activity is positively correlated with the severity of positive symptom scores.^22^ Decreased *AKT1* mRNA level in PBMNCs as assessed by microarray analysis was shown in male, recent-onset (<5 years) patients, which however was not verified by real-time PCR.^46^

Our aims were to explore the TF-miRNA-target gene axis not only for diagnosis purposes but also for treatment monitoring, we therefore selected patients in an actively psychotic state that required immediate medications. It is known that a variety of factors, for example, sampling time, the menstrual cycle, degree of symptoms, medications and so on, affect the measurement of biomarkers.^12, 16^ Many published schizophrenia biomarker studies have not properly recorded these factors.^12, 16^ Meticulous attention was paid to compounding factors in the present study. First, all patients in an acute psychotic state were selected based on their PANSS scores in a way that the severity of symptoms did not vary significantly. Second, all patients had been drug-free for at least 3 months to eliminate drug effects on biomarkers. Third, blood samples were taken in a short timeframe in the early morning (0800–0900 hours), and blood from all female patients was taken ~1 week after a menstrual cycle. We found that *EGR1* was significantly downregulated in PBMNCs from patients. Accordingly, miR-30a-5p and miR-30e-5p were also markedly reduced in patients. Gardiner *et al.*^17^ revealed the decrease of miR-30d and miR-30e in PBMNCs from patients with schizophrenia. They did not report changes in miR-30a-5p levels. The reason for this discrepancy is probably that they used the microarray analysis that is less sensitive than real-time PCR. Interestingly, miR-30a-5p, miR-30b, miR-30d and miR-30e have also been shown to be downregulated in the prefrontal cortex of subjects with schizophrenia compared to healthy subjects.^29^ Decease of miR-30 expression in the brain tissue as well as in PBMNCs suggests that the regulatory machinery for miR-30 expression might be compromised systemically in patients. Of note, Sun *et al.*^19^ reported that miR-30e was increased in PBMNCs from patients with schizophrenia. The reason for this opposing results remains unknown. Further analysis of miR-30a-5p target genes revealed that *NEUROD1* was significantly upregulated in PBMNCs from patients. These data unveiled the alternation of the EGR1-miR-30a-5p-NEUROD1 axis in PBMNCs from patients.

Despite a considerable number of reports demonstrating altered gene/miRNA expression in blood cells from patients with schizophrenia, only a few studies examined the sensitivity and specificity for potential diagnostic utility of the markers.^19, 22, 47^ Lai *et al.*^47^ identified seven altered miRNAs, that is, miR-34a, miR-449a, miR-564, miR-432, miR-548d, miR-572 and miR-652, in PBMNCs isolated from patients. They used this 7-miRNA signature to distinguish the schizophrenia patients from the normal controls and an AUC of 0.93 was determined using the ROC analysis, which is close to ours (0.962 for the EGR1-miR-30a-5p-NEUROD1 axis). Sun *et al.*^19^ revealed an AUC of 0.756 when single miR-30e was employed for schizophrenia diagnosis. We showed an AUC of 0.649 for miR-30a-5p alone. It appears that the use of several miRNAs combined together as did by Lai, or the TF-miRNA-target gene axis as we found in this study, possesses greater diagnostic value than a single miRNA.

In our series, all 30 patients who completed the 12-week treatment exhibited significant improvements. The PANSS total score and the cluster scores for positive symptoms, negative symptoms and general psychopathology symptoms were all significantly reduced. After the 12-week treatment, increased *EGR1* and miR-30a-5p expression but decreased *NEUROD1* expression were observed, suggesting that the EGR1-miR-30a-5p-NEUROD1 axis responded well to the antipsychotics treatment.

To our knowledge, this is the first study that explored the TF-miR-target gene as a potential biomarker for schizophrenia diagnosis and treatment monitoring. Further studies in larger cohorts need to be done to validate the data. Meanwhile, some questions remain to be answered: first, it has been reported that *EGR1* expression is also downregulated in the prefrontal cortex from patients with schizophrenia,^8^ we strongly felt the EGR1-miR-30a-5p-NEUROD1 axis has a role in the pathogenesis of schizophrenia. But what is the role? Second, five second-generation antipsychotics with different pharmacological mechanisms were used, how do these different medications lead to similar changes in the EGR1-miR-30a-5p-NEUROD1 axis? Last, is the EGR1-miR-30a-5p-NEUROD1 axis altered in patients with other psychiatric disorders?

In conclusion, we discovered for the first time that the EGR1-miR-30a-5p-NEUROD1 axis was altered in PBMNCS from schizophrenia patients in acute psychotic state, the axis responded well to antipsychotics treatment, and that the EGR1-miR-30a-5p-NEUROD1 axis possesses greater diagnostic value than the single miR-30a-5p. These data suggest the EGR1-miR-30a-5p-NEUROD1 axis might serve as a promising biomarker for schizophrenia diagnosis and treatment monitoring for those patients in acute psychotic state.

## Figures and Tables

**Table 1:** Primer sequences

*Genes*	*Primer sequence*
*TP53*	F: 5′-CCCTCCTCAGCATCTTATCC-3′
	R: 5′-TGTTCCGTCCCAGTAGATTACC-3′
*ESR2*	F: 5′-GGCACCTTTCTCCTTTAGTGGT-3′
	R: 5′-GCATCCCTCTTTGAACCTGG-3′
*EGR1*	F: 5′-TTTCCCTGAGCCACAAAGC-3′
	R: 5′-TGTGGAAACAGGTAGTCGGG-3′
*BDNF*	F: 5′-AGGCAGGTTCAAGAGGCTT-3′
	R: 5′-TCCAGCAGAAAGAGAAGAGGA-3′
*SMAD1*	F: 5′-CGAATGCCTTAGTGACAGTAGC-3′
	R: 5′-ACATCCTGGCGGTGGTATT-3′
*NEUROD1*	F: 5′-GGTGCCTTGCTATTCTAAGACG-3′
	R: 5′-TGGTGGTGGGTTGGGATAA-3′

Abbreviations: *BDNF*, brain-derived neurotrophic factor; *EGR1*, early growth response protein 1; *ESR2*, estrogen receptor 2; F, forward primer; *NEUROD1*, neurogenic differentiation factor 1; R, reverse primer; *SMAD1*, mothers against decapentaplegic homolog 1; *TP53*, tumor protein 53.

**Table 2:** The demographics of all subjects

*Groups*	*Age (years)*	*Males (%)*	*Year of onset*
Healthy controls	37.0±7.3	18 (36.0%)	
Patients	36.8±10.7	15 (38.9%)	28.4±10.3

Ages were mean±s.d.

**Table 3:** Target genes retrieved from the database multiMiR for miR-30a-5p and miR-30e-5p

*miRNAs*	*Target genes (abbreviation)*
	Forkhead box G1 (*FOXD1*)
	PR domain zinc-finger protein 1 (*PRDM1*)
	Septin 7 (*SEPT7*)
	Trinucleotide repeat containing 6 protein (*TNRC6A*)
	Beclin 1 (*BECN1*)
	Phosphatidylinositol-4,5-bisphosphate 3-Kinase catalytic subunit delta (*PIK3CD*)
	Notch homolog 1 (*NOTCH1*)
miR-30a-5p	**Brain-derived neurotrophic factor (*BDNF*)**
	Denticleless E3 ubiquitin protein ligase homolog (*DTL*)
	Abelson murine leukemia viral oncogene homolog 1 (*ABL1*)
	Runt-related transcription factor 2 (*RUNX2*)
	**Neurogenic differentiation factor 1 (*NEUROD1*)**
	**Mothers against decapentaplegic homolog 1 (*SMAD1*)**
	Apoptosis and caspase activation inhibitor (*AVEN*)
	Tubulin beta 4B class IVb (*TUBB4B*)
miR-30e-5p	Ubiquitin-conjugating enzyme E2I (*UBE2I*)

Highlighted genes were chosen for this study.

**Table 4:** The change of the PANSS scores

	*Before treatment*	*After treatment*	P*-value*
PANSS total score	102.2±15.4	64.4±14.0	<0.001
PANSS positive score	23.4±9.1	10.9±3.6	<0.001
PANSS negative score	24.4± 8.2	18.4±5.8	<0.001
PANSS general psychopathology score	46.2±7.4	31.2±6.5	<0.001

All scores were presented as mean±s.d. from 30 patients who completed the 12-week antipsychotics treatment.
